# Somatosensory and Brainstem Auditory Evoked Potentials Assessed between 4 and 7 Days after Severe Stroke Onset Predict Unfavorable Outcome

**DOI:** 10.1155/2015/196148

**Published:** 2015-12-21

**Authors:** Yan Zhang, Ying Ying Su, Shu Ying Xiao, Yi Fei Liu

**Affiliations:** ^1^Department of Neurology, Xuanwu Hospital, Capital Medical University, Beijing 100053, China; ^2^Department of Neurology, Luhe Hospital, Capital Medical University, Beijing 100053, China

## Abstract

Our objective was to explore the best predictive timing of short-latency somatosensory evoked potentials (SLSEP) and brainstem auditory evoked potentials (BAEP) for unfavorable outcomes in patients with early stage severe stroke. One hundred fifty-six patients with acute severe supratentorial stroke were monitored according to SLSEP, BAEP, and the Glasgow Coma Scale (GCS) at 1–3 days and 4–7 days after the onset of stroke. All patients were followed up for outcomes at 6 months after onset using the modified Rankin Scale (mRS), with a score of 5-6 considered unfavorable. The predictive values of SLSEP, BAEP, and the GCS at 1–3 days were compared with 4–7 days after onset. Our results show that, according to the analysis of prognostic authenticity, the predictive values of SLSEP and BAEP at 4–7 days after stroke onset improved when compared with the values at 1–3 days for unfavorable outcomes. Most of the patients with change of worsening evoked potentials from 1–3 days to 4–7 days after onset had unfavorable outcomes. In conclusion, SLSEP and BAEP assessed at 4–7 days after onset predicted unfavorable outcomes for acute severe stroke patients. The worsening values of SLSEP and BAEP between 1–3 days and 4–7 days also present a prognostic value.

## 1. Introduction

Evoked potentials (EPs) have been widely utilized to predict the outcome of patients with acute severe strokes. Although previous studies have reviewed various timings, that is, within 7 days, 10 to 15 days, 30 days, 10 weeks, and 3 months after stroke onset [1–6], precise timing at early stage remains unclear. Physicians often prefer earlier predictions on prognosis in order to improve treatment strategies. Previous studies have found that short-latency somatosensory evoked potentials (SLSEP) N20 and brainstem auditory evoked potentials (BAEP) wave V within 7 days after stroke were highly consistent with the outcomes of patients [1, 2, 5, 6]. However, 7 days is a relatively long period. Additionally, patients' conditions may change due to brain edemas during the 7-day period after stroke, especially in severe stroke patients. If optimal evaluations can be determined within the 7-day period that accurately predicts outcomes, properly informed and earlier medical treatments can be planned, enabling the optimal use of resources. It is well known that brain edemas take place 3-4 days after stroke, increased intracranial pressure peaks at 4–7 days, and patients often worsen at 4–7 days [7]. We hypothesized that the SLSEP and BAEP could be used to predict more accurate outcomes at 4–7 days than at 1–3 days after the onset of a severe stroke. Accordingly, we conducted a prospective blinded study to test our hypothesis.

## 2. Materials and Methods

### 2.1. Patients

Patients with acute severe supratentorial stroke admitted to the neurointensive care unit (NICU) of Xuanwu Hospital of Capital Medical University between January 2008 and December 2013 were enrolled in the study. The inclusion criteria were the following: (1) age from 18 to 85 years; (2) 1 to 3 days after the onset of the acute severe supratentorial stroke; (3) Glasgow Coma Scale (GCS) [8] ≤12; and (4) severe cerebral infarction or intracranial hemorrhage confirmed by computed tomography and/or magnetic resonance imaging, that is, cerebral infarction volume ≥66% of the middle cerebral artery (MCA) territory [9] and hematoma amount ≥25 mL [10]. The exclusion criteria were the following: (1) presence of severe skin edemas of the upper extremities, cervical or head area; (2) history of severe hearing problems; (3) history of previous stroke; (4) lesions in posterior circulation territory; (5) undergoing intravenous thrombolysis or mechanical thrombectomy therapy; or (6) death within 3 days after the onset. The following parameters were recorded: (1) demographics (age and gender) and (2) stroke presentation, including the baseline National Institutes of Health Stroke Scale (NIHSS) (1–3 days after stroke onset) and the etiology of the brain lesion (infarction or hemorrhage).

### 2.2. Clinical Assessments and Evoked Potentials Monitoring

With the permission of the local ethics committee and the consent of the patients' families, we performed the clinical assessments of GCS and monitored the EPs, including SLSEP and BAEP. The GCS and EPs were examined by two experienced neurologists, respectively, at 1–3 days and 4–7 days after stroke onset. The physician assessing the GCS was blinded to the EPs results. The physician performing the EPs was blinded to the clinical assessments. The EPs were recorded as described previously by Zhang et al. [11] on the electromyography/evoked potential machine (Nicolet Select, Nicolet, Madison, WI, USA) with Ag/AgCl-sintered electrodes.

### 2.3. Clinical Assessments and EPs Parameters

The GCS includes tests of eye opening (1–4 points), speech response (1–5 points), and motor response (1–6 points). The maximum total score is 15 points. A GCS score of 6–12 was defined as a favorable prognostic predictor, and a score of 3–5 was defined as an unfavorable prognostic predictor [12, 13]. The classification of the EPs was defined according to the responses of N20 of SLSEP and V of BAEP: Grade 1, bilaterally normal responses; Grade 2, unilaterally normal responses and pathological responses in the other lateral; Grade 3, bilaterally pathological responses; Grade 4, unilaterally normal responses and absence of responses in the other lateral; Grade 5, unilaterally pathological response and absence of responses in the other lateral; and Grade 6, bilateral absence of responses. Normal EPs limits were defined at 3 SD from the mean value using the normal data bank of our NICU. The unfavorable prognostic predictors of the EPs included any abnormality of responses, that is, Grade 2 to Grade 6, and bilateral absence of responses, that is, Grade 6.

### 2.4. Outcome Evaluation

We assessed outcomes at 6 months because stroke patients may improve following rehabilitation training administered within 6 months after stroke onset. Patients were followed up at 6 months by two physicians who were blinded to the clinical assessments and the EPs results. The modified Rankin Scale (mRS) [14] was utilized to measure outcomes. A score of 0–4 was considered a favorable outcome, whereas a score of 5-6 was graded as an unfavorable one [15]. The optimal outcome achieved within 6 months after stroke was used for analysis.

### 2.5. Statistical Analysis

SPSS version 17.0 was used to analyze the data. The prognosis differences in baseline characteristics and different parameters were assessed with the Mann-Whitney test for continuous variables and Fisher's exact tests for the categorical variables. The authenticity predictions included sensitivity, specificity, positive predictive value (PPV), and negative predictive value (NPV) of the predictors, including GCS 3–5, parameters (N20 was abnormal and bilateral N20 was absent) of SLSEP, and parameters (wave V was abnormal and bilateral wave V was absent) of BAEP for unfavorable outcome. Exact 95% confidence intervals (CIs) were estimated using the binomial distribution. Positive likelihood ratios (LR+) with 95% CIs were also analyzed. If any of the cells of the 2 × 2 table contained no observations, we added a value of 0.5 to each cell to calculate LR+ [16].

## 3. Results

### 3.1. Baseline Data

A total of 156 patients were enrolled of which 94 were men and 62 were women aged between 23 and 85 years (64 ± 14, on average). A total of 123 patients suffered from supratentorial infarction, and 33 patients suffered from cerebral hemispheric hemorrhage. Sixty-seven patients had favorable outcomes, while 89 patients had unfavorable outcomes (Table 1). A total of 111 patients survived.

### 3.2. Prognostic Authenticity Analysis of Possible Predictors for Unfavorable Outcomes at 1–3 Days after Stroke Onset (the First Time)

SLSEP (any abnormality of N20 and bilateral absence of N20) and BAEP (bilateral absence of V) were statistically significant in different outcomes based on Fisher's exact test analysis (*P* < 0.05) at 1–3 days after stroke onset (Table 2). The prognostic authenticity analysis showed that the sensitivity of any abnormality of N20 (96.6%, 95% CI: 89.8%–99.1%) was the best among the clinical assessment of the GCS and EPs. The specificity of bilateral absence of N20 and of bilateral absence of V was the best (100%, 95% CI: 93.2%–100%). The PPV of bilateral absence of N20 (100%, 95% CI: 51.7%–100%) and of bilateral absence of V (100%, 95% CI: 65.5%–100%) was also the best. The NPV of any abnormality of N20 was relatively high (80.0%, 95% CI: 51.4%–94.7%) (Table 3).

### 3.3. Prognostic Authenticity Analysis of Possible Predictors for Unfavorable Outcomes at 4–7 Days after Stroke Onset (the Second Time)

The GCS, SLSEP, and BAEP were all statistically significant in different outcomes based on Fisher's exact test analysis (*P* < 0.05) at 4–7 days after stroke onset (Table 2). The prognostic authenticity analysis showed that the sensitivity of any abnormality of N20 (100%, 95% CI: 94.8%–100%) was the best among the clinical assessment of GCS and EPs. The specificity of bilateral absence of N20 and of bilateral absence of V was the best (100%, 95% CI: 93.2%–100%). The PPV of bilateral absence of N20 (100%, 95% CI: 89.8%–100%) and of bilateral absence of V (100%, 95% CI: 87.4%–100%) was also the best. The NPV of any abnormality of N20 was the best (100%, 95% CI: 78.1%–100%). Furthermore, the bilateral absence of N20 showed the highest LR+ of 65.22 (95% CI: 4.09–1040.76), which was superior to other predictors (Table 3). When compared with 1–3 days after onset, the prognostic authenticity of the GCS, SLSEP, and BAEP improved at 4–7 days.

### 3.4. Analysis of Dynamic Changes of EPs from 1–3 Days to 4–7 Days after Stroke Onset

From 1–3 days to 4–7 days after stroke onset, decreases were noticed with the GCS (from 6–12 to 3–5) in 103 patients; deteriorations were noticed with SLSEP N20 (upgrade, for example, from Grade 2 to Grade 5) in 74 patients with BAEP wave V (upgrade) in 62 patients. These worsening changes were all significant in the different outcomes (*P* < 0.05). Sixty-six of 74 patients (89.2%) with worsening SLSEP and 60 of 62 patients (96.8%) with worsening BAEP ultimately had unfavorable outcomes (Table 4).

## 4. Discussion

We found that SLSEP and BAEP can predict unfavorable outcomes of stroke patients more accurately when assessed at 4–7 days after stroke onset than at 1–3 days. Brain edemas take place 3-4 days after stroke, and increased intracranial pressure peaks at 4–7 days [7]. Patients often worsen during this time period. The predictive timing of acute stroke assessments at 4–7 days after onset is believed to more accurately reflect brain function than assessments at 1–3 days. We also found that SLSEP and BAEP have better predictive accuracy for brain function than GCS.

The absence of N20 indicates an extensive injury of the cerebral cortex and therefore a poor outcome. From our data, the specificity and PPV of bilateral absence of N20 were both 100% within 7 days after stroke onset, which was best among predictors and as good as that of bilateral absence of V of BAEP. SLSEP N20 is generated from a large section of the cortex-subcortex area. After a large number of neurons and/or axes are damaged, N20 will be completely absent. In prior studies, the bilateral loss of SLSEP component N20 in severe brain damage often implied a fatal prognosis in all adult patients (specificity = 93.3% and sensitivity = 59.3%). Only a young child with predominant brainstem hemorrhagic contusion regained consciousness with mild-to-moderate resultant neurological deficits (Glasgow Outcome Scale 3-4) during long-term follow-up of 4 years [17, 18]. We observed that although bilateral N20 emerged in some cases, any N20 abnormality also indicated the possibility of poor outcomes. The sensitivity and NPV of either lateral abnormality of N20 were 100% for unfavorable prognosis.

The bilateral absence of wave V was also a reliable predictor of poor outcomes, especially for bilateral abnormalities. In the present study, the specificity and PPV of the bilateral absence of V were both 100% within 7 days after stroke onset, which was also best among predictors. This is consistent with Liu's findings about patients with putaminal hemorrhage [19]. The BAEP wave V is a robust indicator of brainstem function. If any primary or secondary supratentorial disease deteriorates and impairs the function of brainstem, the V of BAEP originating from the inferior colliculus will first change [6]. Therefore, BAEP wave V is the most reliable predictor for unfavorable outcome of supratentorial stroke.

The worsening of the SLSEP and BAEP between 1–3 days and 4–7 days also provided a prognostic value. Our dynamic assessments found that the GCS, SLSEP, and BAEP worsened in some patients within 7 days after stroke. For more than one-third of 156 patients, the SLSEP and BAEP worsened, and most of these affected patients (89.2–96.8%) had poor outcomes. This indicated that worsening SLSEP and BAEP might be a reliable predictor of the degree of irreversible deterioration. Additionally, the reliability increased with serial recordings. In conclusion, patients with uncertain prognoses 1–3 days after stroke should be further evaluated, especially for the SLSEP and BAEP, to discover the brain function changes for proper and timely clinical decisions [20].

## 5. Conclusions

From this study, 4–7 days after stroke onset was a better timing for predicting outcome, and the SLSEP N20 and BAEP wave V were the most reliable predictors for patients with severe supratentorial strokes. Predictors at 1–3 days after stroke can provide reference values; the worsening of the SLSEP and BAEP between 1–3 days and 4–7 days could provide a strong prognostic value. This study was limited by the small sample size and the use of patients from a single center and could be further expanded by conducting a prospective multicenter study.

## Figures and Tables

**Table 1:** Baseline characteristics and the brain lesions of patients with different outcomes at 6 months after stroke.

	Unfavorable outcome (mRS 5-6)	Favorable outcome (mRS 1–4)	*P* value
Age, median (IQR)	70 (62–76)	60 (47–69)	<0.001
Gender			
Male, *n* (%)	52 (55.3)	42 (44.7)	0.623
Female, *n* (%)	37 (59.7)	25 (40.3)	
NIHSS baseline, median (IQR)	28 (24–32)	27 (22–34)	0.414
Hemisphere stroke			
Left, *n* (%)	47 (55.3)	38 (44.7)	0.745
Right, *n* (%)	42 (59.2)	29 (40.8)	
Etiology of the brain lesion			
Infarction, *n* (%)	73 (59.3)	50 (40.7)	0.323
Hemorrhage, *n* (%)	16 (48.5)	17 (51.5)	
Total, *n* (%)	89 (57.1)	67 (42.9)	

NIHSS, National Institutes of Health Stroke Scale; IQR, interquartile range.

**Table 2:** Prognostic predictors at 1–3 d and 4–7 d after stroke onset with different outcomes at 6 months after stroke.

Prognostic predictors	Unfavorable outcome (mRS 5-6) 1–3 d /4–7 d *n* (%)	Favorable outcome (mRS 1–4) 1–3 d/4–7 d *n* (%)	* P* value 1–3 d/4–7 d
GCS			
3–5	20 (66.7)/56 (81.2)	10 (33.3)/13 (18.8)	0.306/<0.001
6–12	69 (54.8)/33 (37.9)	57 (45.2)/54 (62.1)	

SLSEP			
Abnormality of N20	86 (61.0)/89 (64.5)	55 (39.0)/49 (35.5)	0.004/<0.001
Normality of N20	3 (20.0)/0 (0)	12 (80.0)/18 (100)	

SLSEP			
Bilateral absence of N20	6 (100)/43 (100)	0 (0)/0 (0)	0.037/<0.001
Appearance of N20	83 (55.3)/46 (40.7)	67 (44.7)/67 (59.3)	

BAEP			
Abnormality of V	67 (61.5)/81 (68.1)	42 (38.5)/38 (31.9)	0.113/<0.001
Normality of V	22 (46.8)/8 (21.6)	25 (53.2)/29 (78.4)	

BAEP			
Bilateral absence of V	10 (100)/34 (100)	0 (0)/0 (0)	0.005/<0.001
Appearance of	79 (54.1)/55 (45.1)	67 (45.9)/67 (54.9)	

Total	89 (57.1)	67 (42.9)	

GCS, Glasgow Coma Scale; SLSEP, short-latency somatosensory evoked potential; BAEP, brainstem auditory evoked potential.

**Table 3:** Prognostic authenticity of possible predictors for unfavorable outcomes at 1–3 d and 4–7 d after stroke onset.

Prognostic predictors	Sensitivity % (95% CI) 1–3 d/4–7 d	Specificity % (95% CI) 1–3 d/4–7 d	Positive predictive value % (95% CI) 1–3 d/4–7 d	Negative predictive value % (95% CI) 1–3 d /4–7 d	Positive likelihood ratios % (95% CI) 1–3 d/4–7 d
GCS					
3–5	22.5 (14.6–32.8)/ 62.9 (52.0–72.7)	85.1 (73.8–92.2)/ 80.6 (68.8–88.9)	66.7 (47.1–82.1)/ 81.2 (69.6–89.2)	45.2 (36.4–54.3)/ 62.1 (51.0–72.1)	1.51 (0.76–3.00)/ 3.24 (1.94–5.42)
SLSEP					
Abnormality of N20	96.6 (89.8–99.1)/ 100 (94.8–100)	17.9 (90.0–29.6)/ 26.9 (17.1–39.3)	61.0 (52.4–69.0)/ 64.5 (55.8–72.3)	80.0 (51.4–94.7)/ 100 (78.1–100)	1.18 (1.05–1.33)/ 1.37 (1.18–1.58)
Bilateral absence of N20	6.7 (2.8–14.6)/ 48.3 (37.7–59.1)	100 (93.2–100)/ 100 (93.2–100)	100 (51.7–100)/ 100 (89.8–100)	44.7 (36.6–53.0)/ 59.3 (49.6–68.3)	9.10 (0.52–160.13)/ 65.22 (4.09–1040.76)
BAEP					
Abnormality of V	75.3 (64.8–83.5)/ 91.0 (82.6–95.8)	37.3 (26.1–50.0)/ 43.3 (31.4–55.9)	61.5 (51.6–70.5)/ 68.1 (58.8–76.1)	53.2 (38.2–67.6)/ 78.4 (61.3–89.3)	1.20 (0.96–1.50)/ 1.60 (1.29–2.00)
Bilateral absence of V	11.2 (5.8–20.1)/ 38.2 (28.3–49.2)	100 (93.2–100)/ 100 (93.2–100)	100 (65.5–100)/ 100 (87.4–100)	45.9 (37.7–54.3)/ 54.9 (45.7–63.9)	15.17 (0.91–255.14)/ 51.57 (3.22–826.44)

GCS, Glasgow Coma Scale; SLSEP, short-latency somatosensory evoked potential; BAEP, brainstem auditory evoked potential.

**Table 4:** Analysis of dynamic changes of EPs from 1–3 days to 4–7 d after stroke onset.

Prognostic predictors	Unfavorable outcome (mRS 5-6) *n* (%)	Favorable outcome (mRS 1–4) *n* (%)	*P* value	Sensitivity % (95% CI)	Specificity % (95% CI)	Positive predictive value % (95% CI)	Negative predictive value % (95% CI)	Positive likelihood ratios % (95% CI)
GCS								
Worse (from 6–12 to 3–5)	65 (63.1)	38 (36.9)	0.041	73.0 (62.4–81.6)	26.9 (16.0–41.3)	63.1 (53.0–72.2)	36.8 (22.3–54.0)	1.00 (0.81–1.23)
SLSEP								
Worse (upgrade)	66 (89.2)	8 (10.8)	<0.001	74.2 (63.6–82.6)	88.1 (77.3–94.3)	89.2 (79.3–94.9)	72.0 (60.8–81.0)	6.21 (3.20–12.04)
BAEP								
Worse (upgrade)	60 (96.8)	2 (3.2)	<0.001	67.4 (56.6–76.8)	97.0 (88.7–99.5)	96.8 (87.8–99.4)	69.1 (58.7–78.0)	22.58 (5.72–89.12)

GCS, Glasgow Coma Scale; SLSEP, short-latency somatosensory evoked potential; BAEP, brainstem auditory evoked potential; 95% CI, 95% confidence intervals.
